# Supplementary material for: Revisiting the Concept of Stress in the Prognosis of Solid Tumors: A Role for Stress Granules Proteins?
Source: Cancers (Basel). 2020 Sep 1;12(9):2470. doi: 10.3390/cancers12092470 (PMC7564653; doi:10.3390/cancers12092470)
Supplement: Supplementary file 1 [file cancers-12-02470-s001.pdf]

cancers

Review

Revisiting the Concept of Stress in the Prognosis of Solid Tumors: A Role for Stress Granules Proteins?

Anaïs Aulas, Pascal Finetti, Shawn M. Lyons, François Bertucci, Daniel Birnbaum, Claire Acquaviva, and Emilie Mamessier

Supplementary Materials:

Figure S1

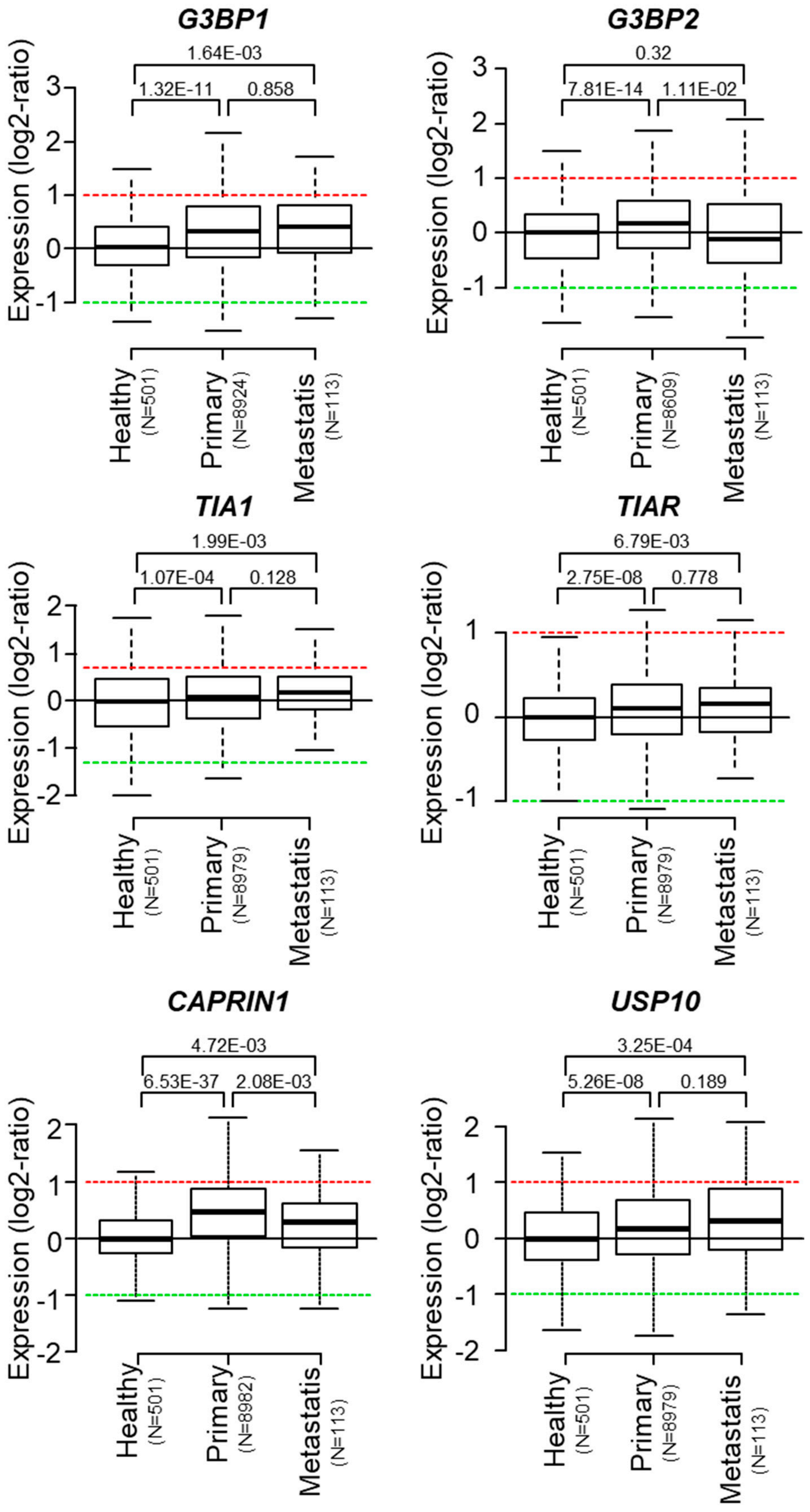
G3BP1
1.64E-03
1.32E-11
0.858
Healthy (N=501)
Primary (N=8924)
Metastatis (N=113)
G3BP2
0.32
7.81E-14
1.11E-02
Healthy (N=501)
Primary (N=8609)
Metastatis (N=113)
TIA1
1.99E-03
1.07E-04
0.128
Healthy (N=501)
Primary (N=8979)
Metastatis (N=113)
TIAR
6.79E-03
2.75E-08
0.778
Healthy (N=501)
Primary (N=8979)
Metastatis (N=113)
CAPRIN1
4.72E-03
6.53E-37
2.08E-03
Healthy (N=501)
Primary (N=8982)
Metastatis (N=113)
USP10
3.25E-04
5.26E-08
0.189
Healthy (N=501)
Primary (N=8979)
Metastatis (N=113)
Expression (log2-ratio)

Figure S1. mRNA expression of SGs genes in breast cancer. SGs genes mRNA expression level (log2) reported as box plots according to normal breast, primary breast cancer, and breast metastatic samples. The *p*-value reported for each comparison was defined by Tukey's range test. The analysis was performed according to supplementary material 1 and Table S1.

Figure S2

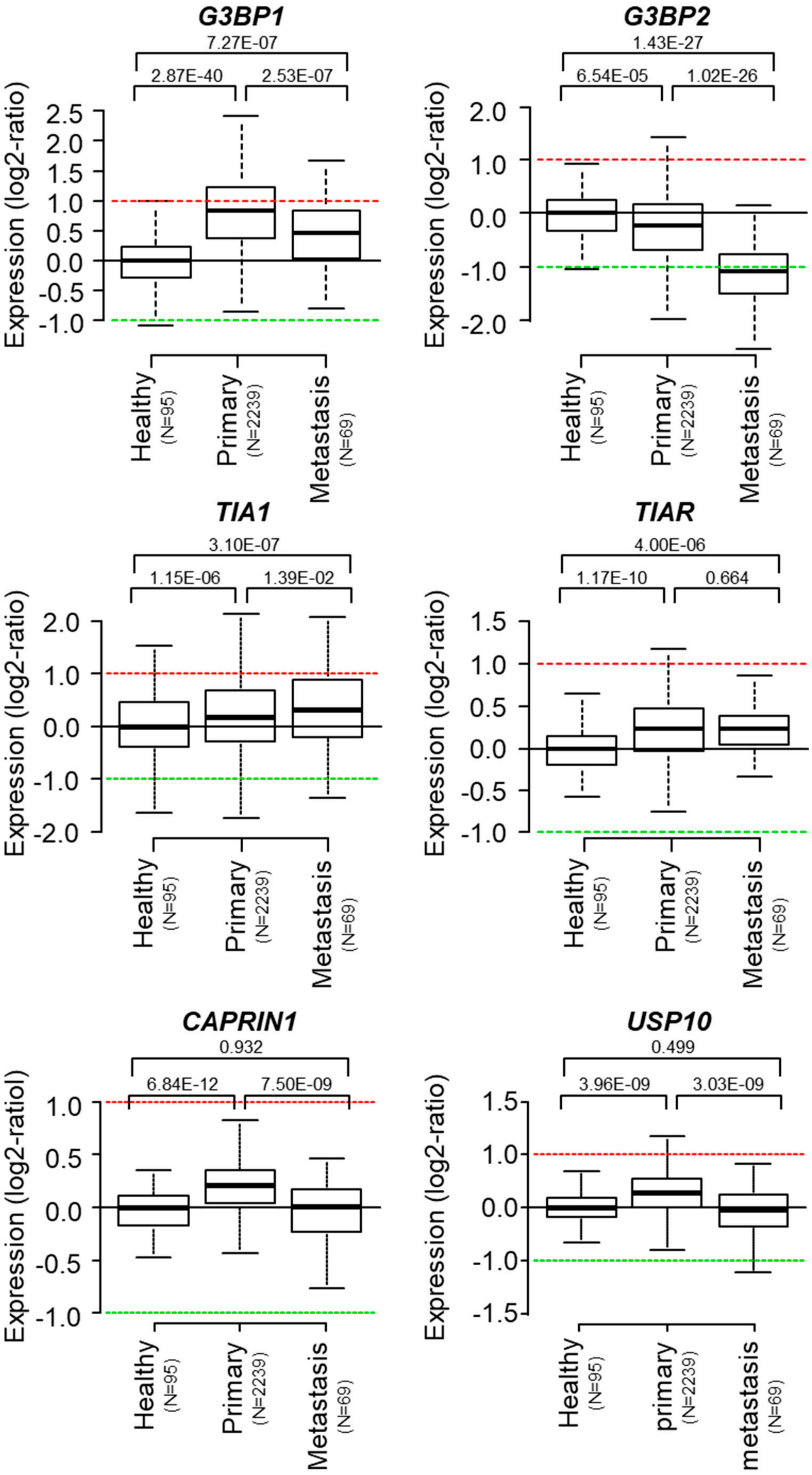

G3BP1
7.27E-07
2.87E-40
2.53E-07
Expression (log2-ratio)
2.5
2.0
1.5
1.0
0.5
0.0
-0.5
-1.0
Healthy (N=95)
Primary (N=2239)
Metastasis (N=69)
G3BP2
1.43E-27
6.54E-05
1.02E-26
Expression (log2-ratio)
2.0
1.0
0.0
-1.0
-2.0
Healthy (N=95)
Primary (N=2239)
Metastasis (N=69)
TIA1
3.10E-07
1.15E-06
1.39E-02
Expression (log2-ratio)
2.0
1.0
0.0
-1.0
-2.0
Healthy (N=95)
Primary (N=2239)
Metastasis (N=69)
TIAR
4.00E-06
1.17E-10
0.664
Expression (log2-ratio)
1.5
1.0
0.5
0.0
-0.5
-1.0
Healthy (N=95)
Primary (N=2239)
Metastasis (N=69)
CAPRIN1
0.932
6.84E-12
7.50E-09
Expression (log2-ratiol)
1.0
0.5
0.0
-0.5
-1.0
Healthy (N=95)
Primary (N=2239)
Metastasis (N=69)
USP10
0.499
3.96E-09
3.03E-09
Expression (log2-ratio)
1.5
1.0
0.0
-1.0
-1.5
Healthy (N=95)
primary (N=2239)
metastasis (N=69)

Figure S2. mRNA expression of SGs genes in colon cancer. SGs genes mRNA expression level (log2) reported as box plots according to normal colon, primary breast cancer, and breast metastatic samples. The *p*-value reported for each comparison was defined by Tukey's range test. The analysis was performed according to supplementary material 1 and Table S1.

Figure S3

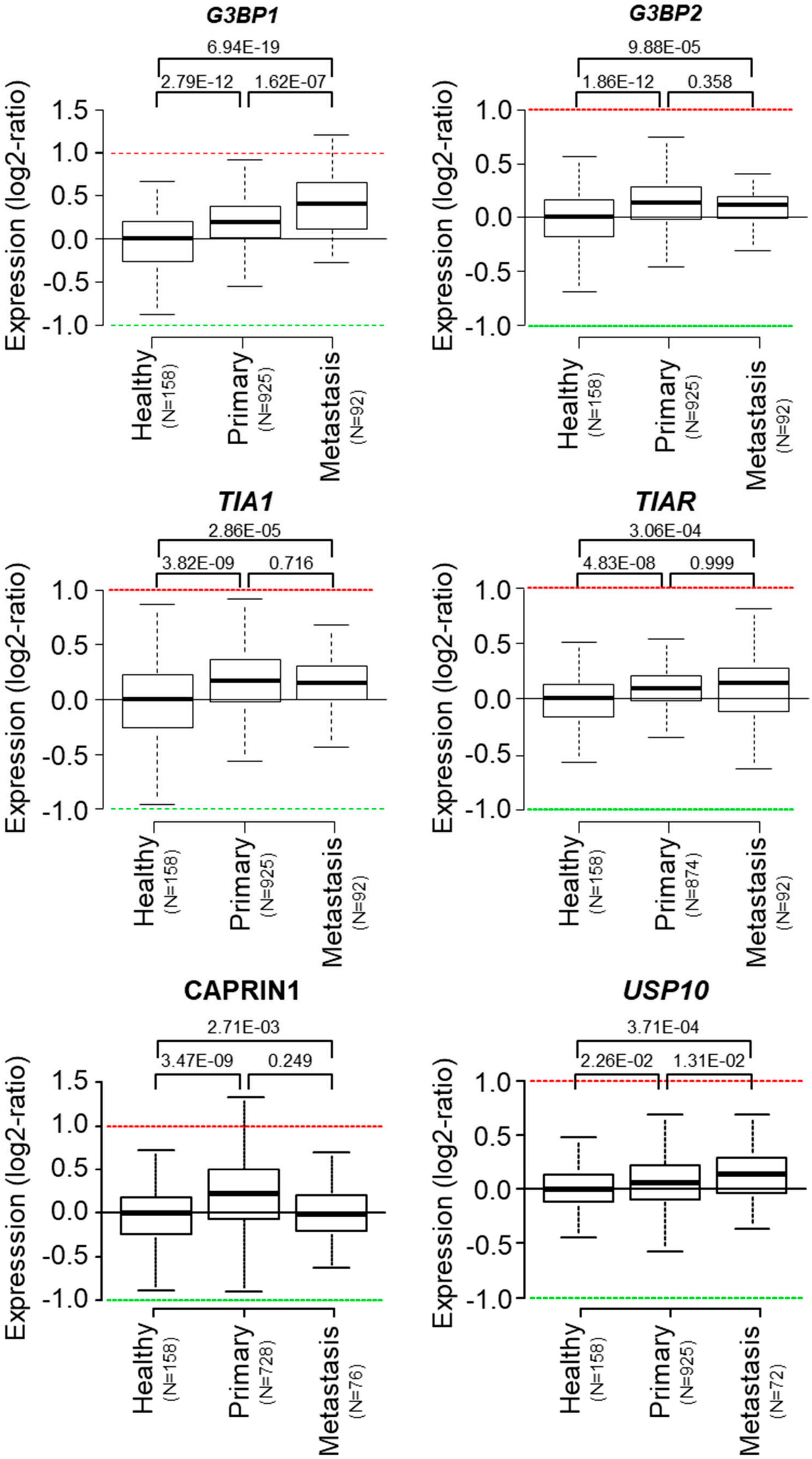
G3BP1
6.94E-19
2.79E-12
1.62E-07
G3BP2
9.88E-05
1.86E-12
0.358
TIA1
2.86E-05
3.82E-09
0.716
TIAR
3.06E-04
4.83E-08
0.999
CAPRIN1
2.71E-03
3.47E-09
0.249
USP10
3.71E-04
2.26E-02
1.31E-02
Expression (log2-ratio)
Expresssion (log2-ratio)
1.5
1.0
0.5
0.0
-0.5
-1.0
Healthy (N=158)
Primary (N=925)
Primary (N=874)
Primary (N=728)
Metastasis (N=92)
Metastasis (N=76)
Metastasis (N=72)

Figure S3: mRNA expression of SG genes in pancreatic cancer. SG genes mRNA expression level (log2) reported as box plots according to normal pancreas, primary breast cancer, and breast metastatic samples. The p-values reported for each comparison was defined by Tukey's range test. The analysis was performed according to supplementary material 1 and Table S1.

Supplementary Material 1: Materials and Methods

Gene Expression Data Sets

We gathered clinicopathological and gene expression data of 35 breast (BR) [1–35], 10 colon (CO) [36–45], and 14 pancreatic (PA) [46–59] cancer data sets comprising at least one probe set representing Stress Granule (Sg) genes i.e. *G3BP1, G3BP2, TIA-1, TIAR, CAPRIN-1 and USP10* (**Table S1**). Data were collected from the National Center for Biotechnology Information (NCBI)/Genbank GEO, ArrayExpress, and TCGA databases. Samples were profiled using whole-genome DNA microarrays (Affymetrix, Agilent, Illumina, homemade) and RNASeq (Illumina). The pooled data set contained 9,885 BR cancer samples, including 8,982 primary cancers, 113 metastases, and 501 normal samples, 2,308 CO cancer samples, including 2,239 primary cancers, 69 metastases and 95 normal samples, and 1,017 PA cancer samples, including 925 primary, 92 metastases, and 158 normal samples. The study was approved by our institutional board.

Gene Expression Data Analysis

Data analysis required pre-analytic processing. First, we normalized each DNA microarray-based data set separately, by using quantile normalization for the available processed data (Agilent, Illumina and homemade microarray), and Robust Multichip Average (RMA) with the non-parametric quantile algorithm for the raw Affymetrix data sets. Normalization was done in R using Bioconductor and associated packages. Then, we mapped hybridization probes across the different technological platforms present. We used SOURCE (http://smd.stanford. edu/cgi-bin/source/sourceSearch) and EntrezGene (Homo sapiens gene information db, release from 04/27/2017), ftp://ftp.ncbi.nlm.nih.gov/gene/) to retrieve and update the Agilent annotations, and NetAffx Annotation files (www.affymetrix.com; release from 01/12/2008) for the Affymetrix annotations. The probes were then mapped according to their EntrezGeneID, and when multiple probes represented the same GeneID, we retained the one with the highest variance in a particular dataset. For the RNA-seq data, we used the available normalized RNASeq data that we log_2-transformed. Next, we corrected the studies for batch effects using z-score normalization. Briefly, for each Sg gene expression value in each study separately, the value was transformed by subtracting the mean of the gene in that dataset divided by its standard deviation, mean and standard deviation being measured on primary cancer samples only.

Statistical Analysis

The correlation between Sg genes and sample type was defined by Tukey's range test. Overall survival was calculated from the date of diagnosis to the date of death from cancer, Metastasis-free survival was calculated from the date of diagnosis to the date of the distant relapse, and disease-free survival was calculated from the date of diagnosis to the date of the relapse or death from cancer. Follow-up was measured from the date of diagnosis to the date of last news for event-free patients. Survival analyses were done using Cox regression analysis (Wald test). All statistical tests were two-sided at the 5% level of significance. Statistical analysis was done using the survival package (version 3.1-12) in the R software (version 3.5.2; http://www.cran.r-project.org/).

Table S1. List of data sets included in the study.

Reference	Cancer Type	Source of Data
van de Vijver et al., NEJM 2002	Breast Cancer	https://ccb.nki.nl/data/ZipFiles295Samples.zip
van't Veer et al., Nature 2002	Breast Cancer	https://ccb.nki.nl/data/van-t-Veer_Nature_2002/
Farmer P et al., Oncogene 2005	Breast Cancer	GEO: GSE1561
Minn AJ et al., Nature 2005	Breast Cancer	GEO: GSE2603
Wang Y et al., Lancet 2005	Breast Cancer	GEO: GSE2034
Hess KR et al., J Clin Oncol 2006	Breast Cancer	MDA133
Ivshina et al., Cancer Res 2006	Breast Cancer	GEO: GSE4922, GSE1456
Sotiriou C et al., J Natl Cancer Inst 2006	Breast Cancer	GEO: GSE2990
Bonnefoi et al., Lancet Oncol 2007	Breast Cancer	GEO: GSE6861, GSE4779
Desmedt C etal., Clin Cancer Res 2007	Breast Cancer	GEO: GSE7390
Miller WR et al., Breast Cancer Res 2010	Breast Cancer	GEO: GSE5462
Klein A et al., Int J Cancer 2007	Breast Cancer	GEO: GSE6596
Bos et al., Nature 2009	Breast Cancer	GEO: GSE12276
Hoeflich et al., Clin Cancer Res 2009	Breast Cancer	GEO: GSE12763
Marty et al., Breast Cancer Res 2008	Breast Cancer	GEO: GSE13787
Merritt WM et al., N Engl J Med 2008	Breast Cancer	Array Express: E-MTAB-158
Schmidt M etal., Cancer Res 2008	Breast Cancer	GEO: GSE11121
Yu K et al., PLoS Genet 2008	Breast Cancer	GEO: GSE5364
Zhang Y et al., Breast Cancer Res Treat 2009	Breast Cancer	GEO: GSE12093
Barry et al., J Clin Oncol 2010	Breast Cancer	GEO: GSE23593
Iwamoto T et al., J Natl Cancer Inst 2011	Breast Cancer	GEO: GSE22093, GSE22597

Korde et al.,<br>Breast Cancer Res Treat 2010	Breast Cancer	GEO: GSE18728
Prat A et al.,<br>Breast Cancer Res 2010	Breast Cancer	GEO: GSE18229
Silver et al.,<br>J Clin Oncol 2010	Breast Cancer	GEO: GSE18864
Tabchy A et al.,<br>Clin Cancer Res 2010	Breast Cancer	GEO: GSE20271
Jonsson et al.,<br>BCR 2010	Breast Cancer	GEO: GSE22133
Chen et al.,<br>Breast Cancer Res Treat 2010	Breast Cancer	GEO: GSE10780
Desmedt et al.,<br>J Clin Oncol 2011	Breast Cancer	GEO: GSE16446
Guedj et al.,<br>Oncogene 2011	Breast Cancer	Array Express: E-MTAB-365
Hatzis C et al.,<br>JAMA 2011	Breast Cancer	GEO: GSE25066
Popovici V et al.,<br>Breast Cancer Res 2010	Breast Cancer	GEO: GSE20194
TCGA,<br>Nature 2012	Breast Cancer	TCGA Data Portal - BRCA -
Ellis et al.,<br>Nature 2012	Breast Cancer	GEO: GSE29442, GSE35186
Curtis et al.,<br>Nature 2012	Breast Cancer	EGA: EGAS00000000083
Sabatier R et al., (our IPC series)<br>PLoS One 2011	Breast Cancer	GEO: GSE31448
Jorissen et al.,<br>Clin Cancer Res 2009	Colon Cancer	GEO: GSE14333
Sheffer et al.,<br>Proc Natl Acad Sci 2009	Colon Cancer	GEO: GSE41258
Staub et al.,<br>J Mol Med (Berl) 2009	Colon Cancer	GEO: GSE12945
Smith et al.,<br>Gastroenterology 2010	Colon Cancer	GEO: GSE17538
de Sousa et al.,<br>Cell Stem Cell 2011	Colon Cancer	GEO: GSE33113
Kenned et al.,<br>J Clin Oncol 2011	Colon Cancer	Array-Express: E-MTAB-863
Sveen et al.,<br>Genome Med 2011	Colon Cancer	GEO: GSE24551
Laibe et al.,<br>OMICS 2012	Colon Cancer	GEO: GSE37892

Marisa et al., PLoS Med 2013	Colon Cancer	GEO: GSE39582
TCGA, COAD	Colon Cancer	TCGA portal, https://tcga-data.nci.nih.gov
Badea et al., Hepatogastroenterology 2007	Pancreatic Cancer	GEO: GSE15471
van den Broeck et al., J Exp Clin Cancer Res 2012	Pancreatic Cancer	GEO: GSE42952
Zhang et al., PLoS One 2012 & CCR2013	Pancreatic Cancer	GEO: GSE28735
Lunardi et al., Oncotarget 2014	Pancreatic Cancer	GEO: GSE55643
Park et al., Mod Pathol 2014	Pancreatic Cancer	GEO: GSE43795
Winter et al., PLoS Computational Biology 2012	Pancreatic Cancer	Array-Express: E-MEXP-2780
Grutzmann et al., Neoplasia 2004	Pancreatic Cancer	Array-Express: E-MEXP-950
TCGA, PAAD	Pancreatic Cancer	TCGA Data Portal - COAD -
Straford et al., PLoS Med 2010	Pancreatic Cancer	GEO: GSE21501
Monzon et al., Clin Oncol 2009	Pancreatic Cancer	GEO: GSE12630
Bailey et al., Nature 2016	Pancreatic Cancer	EGA: EGAS00001000154
Chen et al., PLoS ONE 2015	Pancreatic Cancer	GEO: GSE57495
Collisson et al., Nat Med. 2011	Pancreatic Cancer	GEO: GSE17891
Kirby et al., Mol Oncol. 2016	Pancreatic Cancer	GEO: GSE79670

References

1. van de Vijver, M.J.; He, Y.D.; van't Veer, L.J.; Dai, H.; Hart, A.A.; Voskuil, D.W.; Schreiber, G.J.; Peterse, J.L.; Roberts, C.; Marton, M.J.; et al. A gene-expression signature as a predictor of survival in breast cancer. *N. Engl. J. Med.* **2002**, *347*, 1999–2009, doi:10.1056/NEJMoa021967.
2. van 't Veer, L.J.; Dai, H.; van de Vijver, M.J.; He, Y.D.; Hart, A.A.; Mao, M.; Peterse, H.L.; van der Kooy, K.; Marton, M.J.; Witteveen, A.T.; et al. Gene expression profiling predicts clinical outcome of breast cancer. *Nature* **2002**, *415*, 530–536, doi:10.1038/415530a.
3. Farmer, P.; Bonnefoi, H.; Becette, V.; Tubiana-Hulin, M.; Fumoleau, P.; Larsimont, D.; Macgrogan, G.; Bergh, J.; Cameron, D.; Goldstein, D.; et al. Identification of molecular apocrine breast tumours by microarray analysis. *Oncogene* **2005**, *24*, 4660–4671, doi:10.1038/sj.onc.1208561.
4. Minn, A.J.; Gupta, G.P.; Siegel, P.M.; Bos, P.D.; Shu, W.; Giri, D.D.; Viale, A.; Olshen, A.B.; Gerald, W.L.; Massague, J. Genes that mediate breast cancer metastasis to lung. *Nature* **2005**, *436*, 518–524, doi:10.1038/nature03799.

5. Wang, Y.; Klijn, J.G.; Zhang, Y.; Sieuwerts, A.M.; Look, M.P.; Yang, F.; Talantov, D.; Timmermans, M.; Meijer-van Gelder, M.E.; Yu, J.; et al. Gene-expression profiles to predict distant metastasis of lymph-node-negative primary breast cancer. *Lancet* **2005**, *365*, 671–679, doi:10.1016/S0140-6736(05)17947-1.
6. Hess, K.R.; Anderson, K.; Symmans, W.F.; Valero, V.; Ibrahim, N.; Mejia, J.A.; Booser, D.; Theriault, R.L.; Buzdar, A.U.; Dempsey, P.J.; et al. Pharmacogenomic predictor of sensitivity to preoperative chemotherapy with paclitaxel and fluorouracil, doxorubicin, and cyclophosphamide in breast cancer. *J. Clin. Oncol.* **2006**, *24*, 4236–4244, doi:10.1200/JCO.2006.05.6861.
7. Ivshina, A.V.; George, J.; Senko, O.; Mow, B.; Putti, T.C.; Smeds, J.; Lindahl, T.; Pawitan, Y.; Hall, P.; Nordgren, H.; et al. Genetic reclassification of histologic grade delineates new clinical subtypes of breast cancer. *Cancer Res.* **2006**, *66*, 10292–10301, doi:10.1158/0008-5472.CAN-05-4414.
8. Sotiriou, C.; Wirapati, P.; Loi, S.; Harris, A.; Fox, S.; Smeds, J.; Nordgren, H.; Farmer, P.; Praz, V.; Haibe-Kains, B.; et al. Gene expression profiling in breast cancer: Understanding the molecular basis of histologic grade to improve prognosis. *J. Natl. Cancer Inst.* **2006**, *98*, 262–272, doi:10.1093/jnci/djj052.
9. Bonnefoi, H.; Potti, A.; Delorenzi, M.; Mauriac, L.; Campone, M.; Tubiana-Hulin, M.; Petit, T.; Rouanet, P.; Jassem, J.; Blot, E.; et al. Validation of gene signatures that predict the response of breast cancer to neoadjuvant chemotherapy: A substudy of the EORTC 10994/BIG 00-01 clinical trial. *Lancet Oncol.* **2007**, *8*, 1071–1078, doi:10.1016/S1470-2045(07)70345-5.
10. Desmedt, C.; Piette, F.; Loi, S.; Wang, Y.; Lallemand, F.; Haibe-Kains, B.; Viale, G.; Delorenzi, M.; Zhang, Y.; d'Assignies, M.S.; et al. Strong time dependence of the 76-gene prognostic signature for node-negative breast cancer patients in the TRANSBIG multicenter independent validation series. *Clin. Cancer Res.* **2007**, *13*, 3207–3214, doi:10.1158/1078-0432.CCR-06-2765.
11. Miller, W.R.; Larionov, A. Changes in expression of oestrogen regulated and proliferation genes with neoadjuvant treatment highlight heterogeneity of clinical resistance to the aromatase inhibitor, letrozole. *Breast Cancer Res.* **2010**, *12*, R52, doi:10.1186/bcr2611.
12. Klein, A.; Wessel, R.; Graessmann, M.; Jurgens, M.; Petersen, I.; Schmutzler, R.; Niederacher, D.; Arnold, N.; Meindl, A.; Scherneck, S.; et al. Comparison of gene expression data from human and mouse breast cancers: Identification of a conserved breast tumor gene set. *Int. J. Cancer* **2007**, *121*, 683–688, doi:10.1002/ijc.22630.
13. Bos, P.D.; Zhang, X.H.; Nadal, C.; Shu, W.; Gomis, R.R.; Nguyen, D.X.; Minn, A.J.; van de Vijver, M.J.; Gerald, W.L.; Foekens, J.A.; et al. Genes that mediate breast cancer metastasis to the brain. *Nature* **2009**, *459*, 1005–1009, doi:10.1038/nature08021.
14. Hoeflich, K.P.; O'Brien, C.; Boyd, Z.; Cavet, G.; Guerrero, S.; Jung, K.; Januario, T.; Savage, H.; Punnoose, E.; Truong, T.; et al. In vivo antitumor activity of MEK and phosphatidylinositol 3-kinase inhibitors in basal-like breast cancer models. *Clin. Cancer Res.* **2009**, *15*, 4649–4664, doi:10.1158/1078-0432.CCR-09-0317.
15. Marty, B.; Maire, V.; Gravier, E.; Rigaill, G.; Vincent-Salomon, A.; Kappler, M.; Lebigot, I.; Djelti, F.; Tourdes, A.; Gestraud, P.; et al. Frequent PTEN genomic alterations and activated phosphatidylinositol 3-kinase pathway in basal-like breast cancer cells. *Breast Cancer Res.* **2008**, *10*, R101, doi:10.1186/bcr2204.
16. Merritt, W.M.; Lin, Y.G.; Han, L.Y.; Kamat, A.A.; Spannuth, W.A.; Schmandt, R.; Urbauer, D.; Pennacchio, L.A.; Cheng, J.F.; Nick, A.M.; et al. Dicer, Drosha, and outcomes in patients with ovarian cancer. *N. Engl. J. Med.* **2008**, *359*, 2641–2650, doi:10.1056/NEJMoa0803785.
17. Schmidt, M.; Bohm, D.; von Torne, C.; Steiner, E.; Puhl, A.; Pilch, H.; Lehr, H.A.; Hengstler, J.G.; Kolbl, H.; Gehrmann, M. The humoral immune system has a key prognostic impact in node-negative breast cancer. *Cancer Res.* **2008**, *68*, 5405–5413, doi:10.1158/0008-5472.CAN-07-5206.
18. Yu, K.; Ganesan, K.; Tan, L.K.; Laban, M.; Wu, J.; Zhao, X.D.; Li, H.; Leung, C.H.; Zhu, Y.; Wei, C.L.; et al. A precisely regulated gene expression cassette potently modulates metastasis and survival in multiple solid cancers. *PLoS Genet.* **2008**, *4*, e1000129, doi:10.1371/journal.pgen.1000129.
19. Barry, W.T.; Kernagis, D.N.; Dressman, H.K.; Griffis, R.J.; Hunter, J.D.; Olson, J.A.; Marks, J.R.; Ginsburg, G.S.; Marcom, P.K.; Nevins, J.R.; et al. Intratumor heterogeneity and precision of microarray-based predictors of breast cancer biology and clinical outcome. *J. Clin. Oncol.* **2010**, *28*, 2198–2206, doi:10.1200/JCO.2009.26.7245.
20. Iwamoto, T.; Bianchini, G.; Booser, D.; Qi, Y.; Coutant, C.; Shiang, C.Y.; Santarpia, L.; Matsuoka, J.; Hortobagyi, G.N.; Symmans, W.F.; et al. Gene pathways associated with prognosis and chemotherapy sensitivity in molecular subtypes of breast cancer. *J. Natl. Cancer Inst.* **2011**, *103*, 264–272, doi:10.1093/jnci/djq524.

21. Korde, L.A.; Lusa, L.; McShane, L.; Lebowitz, P.F.; Lukes, L.; Camphausen, K.; Parker, J.S.; Swain, S.M.; Hunter, K.; Zujewski, J.A. Gene expression pathway analysis to predict response to neoadjuvant docetaxel and capecitabine for breast cancer. *Breast Cancer Res. Treat.* **2010**, *119*, 685–699, doi:10.1007/s10549-009-0651-3.
22. Prat, A.; Parker, J.S.; Karginova, O.; Fan, C.; Livasy, C.; Herschkowitz, J.I.; He, X.; Perou, C.M. Phenotypic and molecular characterization of the claudin-low intrinsic subtype of breast cancer. *Breast Cancer Res.* **2010**, *12*, R68, doi:10.1186/bcr2635.
23. Silver, D.P.; Richardson, A.L.; Eklund, A.C.; Wang, Z.C.; Szallasi, Z.; Li, Q.; Juul, N.; Leong, C.O.; Calogrias, D.; Buraimoh, A.; et al. Efficacy of neoadjuvant Cisplatin in triple-negative breast cancer. *J. Clin. Oncol.* **2010**, *28*, 1145–1153, doi:10.1200/JCO.2009.22.4725.
24. Tabchy, A.; Valero, V.; Vidaurre, T.; Lluch, A.; Gomez, H.; Martin, M.; Qi, Y.; Barajas-Figueroa, L.J.; Souchon, E.; Coutant, C.; et al. Evaluation of a 30-gene paclitaxel, fluorouracil, doxorubicin, and cyclophosphamide chemotherapy response predictor in a multicenter randomized trial in breast cancer. *Clin. Cancer Res.* **2010**, *16*, 5351–5361, doi:10.1158/1078-0432.CCR-10-1265.
25. Jonsson, S.; Olsson, B.; Jacobsson, S.; Palmqvist, L.; Ricksten, A.; Ekeland-Sjoberg, K.; Wadenvik, H. BCR-ABL1 transcript levels increase in peripheral blood but not in granulocytes after physical exercise in patients with chronic myeloid leukemia. *Scand. J. Clin. Lab. Investig.* **2011**, *71*, 7–11, doi:10.3109/00365513.2010.521981.
26. Desmedt, C.; Di Leo, A.; de Azambuja, E.; Larsimont, D.; Haibe-Kains, B.; Selleslags, J.; Delaloge, S.; Duhem, C.; Kains, J.P.; Carly, B.; et al. Multifactorial approach to predicting resistance to anthracyclines. *J. Clin. Oncol.* **2011**, *29*, 1578–1586, doi:10.1200/JCO.2010.31.2231.
27. Guedj, M.; Marisa, L.; de Reynies, A.; Orsetti, B.; Schiappa, R.; Bibeau, F.; MacGrogan, G.; Lerebours, F.; Finetti, P.; Longy, M.; et al. A refined molecular taxonomy of breast cancer. *Oncogene* **2012**, *31*, 1196–1206, doi:10.1038/onc.2011.301.
28. Hatzis, C.; Pusztai, L.; Valero, V.; Booser, D.J.; Esserman, L.; Lluch, A.; Vidaurre, T.; Holmes, F.; Souchon, E.; Wang, H.; et al. A genomic predictor of response and survival following taxane-anthracycline chemotherapy for invasive breast cancer. *JAMA* **2011**, *305*, 1873–1881, doi:10.1001/jama.2011.593.
29. Popovici, V.; Chen, W.; Gallas, B.G.; Hatzis, C.; Shi, W.; Samuelson, F.W.; Nikolsky, Y.; Tsyganova, M.; Ishkin, A.; Nikolskaya, T.; et al. Effect of training-sample size and classification difficulty on the accuracy of genomic predictors. *Breast Cancer Res.* **2010**, *12*, R5, doi:10.1186/bcr2468.
30. Ellis, M.J.; Ding, L.; Shen, D.; Luo, J.; Suman, V.J.; Wallis, J.W.; Van Tine, B.A.; Hoog, J.; Goiffon, R.J.; Goldstein, T.C.; et al. Whole-genome analysis informs breast cancer response to aromatase inhibition. *Nature* **2012**, *486*, 353–360, doi:10.1038/nature11143.
31. Curtis, C.; Shah, S.P.; Chin, S.F.; Turashvili, G.; Rueda, O.M.; Dunning, M.J.; Speed, D.; Lynch, A.G.; Samarajiwa, S.; Yuan, Y.; et al. The genomic and transcriptomic architecture of 2,000 breast tumours reveals novel subgroups. *Nature* **2012**, *486*, 346–352, doi:10.1038/nature10983.
32. Sabatier, R.; Finetti, P.; Adelaide, J.; Guille, A.; Borg, J.P.; Chaffanet, M.; Lane, L.; Birnbaum, D.; Bertucci, F. Down-regulation of ECRG4, a candidate tumor suppressor gene, in human breast cancer. *PLoS ONE* **2011**, *6*, e27656, doi:10.1371/journal.pone.0027656.
33. Zhang, Y.; Sieuwerts, A.M.; McGreevy, M.; Casey, G.; Cufer, T.; Paradiso, A.; Harbeck, N.; Span, P.N.; Hicks, D.G.; Crowe, J.; et al. The 76-gene signature defines high-risk patients that benefit from adjuvant tamoxifen therapy. *Breast Cancer Res. Treat.* **2009**, *116*, 303–309, doi:10.1007/s10549-008-0183-2.
34. Chen, D.T.; Nasir, A.; Culhane, A.; Venkataramu, C.; Fulp, W.; Rubio, R.; Wang, T.; Agrawal, D.; McCarthy, S.M.; Gruidl, M.; et al. Proliferative genes dominate malignancy-risk gene signature in histologically-normal breast tissue. *Breast Cancer Res. Treat.* **2010**, *119*, 335–346, doi:10.1007/s10549-009-0344-y.
35. Cancer Genome Atlas, N. Comprehensive molecular portraits of human breast tumours. *Nature* **2012**, *490*, 61–70, doi:10.1038/nature11412.
36. Jorissen, R.N.; Gibbs, P.; Christie, M.; Prakash, S.; Lipton, L.; Desai, J.; Kerr, D.; Aaltonen, L.A.; Arango, D.; Kruhoffer, M.; et al. Metastasis-Associated Gene Expression Changes Predict Poor Outcomes in Patients with Dukes Stage B and C Colorectal Cancer. *Clin. Cancer Res.* **2009**, *15*, 7642–7651, doi:10.1158/1078-0432.CCR-09-1431.
37. Sheffer, M.; Bacolod, M.D.; Zuk, O.; Giardina, S.F.; Pincas, H.; Barany, F.; Paty, P.B.; Gerald, W.L.; Notterman, D.A.; Domany, E. Association of survival and disease progression with chromosomal

instability: A genomic exploration of colorectal cancer. *Proc. Natl. Acad. Sci. USA* **2009**, *106*, 7131–7136, doi:10.1073/pnas.0902232106.
38. Staub, E.; Groene, J.; Heinze, M.; Mennerich, D.; Roepcke, S.; Klaman, I.; Hinzmann, B.; Castanos-Velez, E.; Pilarsky, C.; Mann, B.; et al. An expression module of WIPF1-coexpressed genes identifies patients with favorable prognosis in three tumor types. *J. Mol. Med.* **2009**, *87*, 633–644, doi:10.1007/s00109-009-0467-y.
39. Smith, J.J.; Deane, N.G.; Wu, F.; Merchant, N.B.; Zhang, B.; Jiang, A.; Lu, P.; Johnson, J.C.; Schmidt, C.; Bailey, C.E.; et al. Experimentally derived metastasis gene expression profile predicts recurrence and death in patients with colon cancer. *Gastroenterology* **2010**, *138*, 958–968, doi:10.1053/j.gastro.2009.11.005.
40. Kennedy, R.D.; Bylesjo, M.; Kerr, P.; Davison, T.; Black, J.M.; Kay, E.W.; Holt, R.J.; Proutski, V.; Ahdesmaki, M.; Farztdinov, V.; et al. Development and independent validation of a prognostic assay for stage II colon cancer using formalin-fixed paraffin-embedded tissue. *J. Clin. Oncol.* **2011**, *29*, 4620–4626, doi:10.1200/JCO.2011.35.4498.
41. Sveen, A.; Agesen, T.H.; Nesbakken, A.; Rognum, T.O.; Lothe, R.A.; Skotheim, R.I. Transcriptome instability in colorectal cancer identified by exon microarray analyses: Associations with splicing factor expression levels and patient survival. *Genome Med.* **2011**, *3*, 32, doi:10.1186/gm248.
42. Laibe, S.; Lagarde, A.; Ferrari, A.; Monges, G.; Birnbaum, D.; Olschwang, S.; Project, C.O.L. A seven-gene signature aggregates a subgroup of stage II colon cancers with stage III. *OMICS* **2012**, *16*, 560–565, doi:10.1089/omi.2012.0039.
43. Marisa, L.; de Reynies, A.; Duval, A.; Selves, J.; Gaub, M.P.; Vescovo, L.; Etienne-Grimaldi, M.C.; Schiappa, R.; Guenot, D.; Ayadi, M.; et al. Gene expression classification of colon cancer into molecular subtypes: Characterization, validation, and prognostic value. *PLoS Med.* **2013**, *10*, e1001453, doi:10.1371/journal.pmed.1001453.
44. de Sousa, E.M.F.; Colak, S.; Buikhuisen, J.; Koster, J.; Cameron, K.; de Jong, J.H.; Tuynman, J.B.; Prasetyanti, P.R.; Fessler, E.; van den Bergh, S.P.; et al. Methylation of cancer-stem-cell-associated Wnt target genes predicts poor prognosis in colorectal cancer patients. *Cell Stem Cell* **2011**, *9*, 476–485, doi:10.1016/j.stem.2011.10.008.
45. Cancer Genome Atlas, N. Comprehensive molecular characterization of human colon and rectal cancer. *Nature* **2012**, *487*, 330–337, doi:10.1038/nature11252.
46. Badea, R.; Socaciu, M.; Lupsor, M.; Mosteanu, O.; Pop, T. Evaluating the liver tumors using three-dimensional ultrasonography. A pictorial essay. *J. Gastrointestin Liver Dis.* **2007**, *16*, 85–92.
47. Van den Broeck, A.; Vankelecom, H.; Van Eijsden, R.; Govaere, O.; Topal, B. Molecular markers associated with outcome and metastasis in human pancreatic cancer. *J. Exp. Clin. Cancer Res.* **2012**, *31*, 68, doi:10.1186/1756-9966-31-68.
48. Lunardi, S.; Jamieson, N.B.; Lim, S.Y.; Griffiths, K.L.; Carvalho-Gaspar, M.; Al-Assar, O.; Yameen, S.; Carter, R.C.; McKay, C.J.; Spoletini, G.; et al. IP-10/CXCL10 induction in human pancreatic cancer stroma influences lymphocytes recruitment and correlates with poor survival. *Oncotarget* **2014**, *5*, 11064–11080, doi:10.18632/oncotarget.2519.
49. Park, M.; Kim, M.; Hwang, D.; Park, M.; Kim, W.K.; Kim, S.K.; Shin, J.; Park, E.S.; Kang, C.M.; Paik, Y.K.; et al. Characterization of gene expression and activated signaling pathways in solid-pseudopapillary neoplasm of pancreas. *Mod. Pathol.* **2014**, *27*, 580–593, doi:10.1038/modpathol.2013.154.
50. Grutzmann, R.; Saeger, H.D.; Luttges, J.; Schackert, H.K.; Kalthoff, H.; Kloppel, G.; Pilarsky, C. Microarray-based gene expression profiling in pancreatic ductal carcinoma: Status quo and perspectives. *Int. J. Colorectal Dis.* **2004**, *19*, 401–413, doi:10.1007/s00384-003-0563-3.
51. Monzon, F.A.; Lyons-Weiler, M.; Buturovic, L.J.; Rigl, C.T.; Henner, W.D.; Sciulli, C.; Dumur, C.I.; Medeiros, F.; Anderson, G.G. Multicenter validation of a 1,550-gene expression profile for identification of tumor tissue of origin. *J. Clin. Oncol.* **2009**, *27*, 2503–2508, doi:10.1200/JCO.2008.17.9762.
52. Bailey, P.; Chang, D.K.; Nones, K.; Johns, A.L.; Patch, A.M.; Gingras, M.C.; Miller, D.K.; Christ, A.N.; Bruxner, T.J.; Quinn, M.C.; et al. Genomic analyses identify molecular subtypes of pancreatic cancer. *Nature* **2016**, *531*, 47–52, doi:10.1038/nature16965.
53. Collisson, E.A.; Sadanandam, A.; Olson, P.; Gibb, W.J.; Truitt, M.; Gu, S.; Cooc, J.; Weinkle, J.; Kim, G.E.; Jakkula, L.; et al. Subtypes of pancreatic ductal adenocarcinoma and their differing responses to therapy. *Nat. Med.* **2011**, *17*, 500–503, doi:10.1038/nm.2344.
54. Kirby, M.K.; Ramaker, R.C.; Gertz, J.; Davis, N.S.; Johnston, B.E.; Oliver, P.G.; Sexton, K.C.; Greeno, E.W.; Christein, J.D.; Heslin, M.J.; et al. RNA sequencing of pancreatic adenocarcinoma tumors yields

novel expression patterns associated with long-term survival and reveals a role for ANGPTL4. *Mol. Oncol.* **2016**, *10*, 1169–1182, doi:10.1016/j.molonc.2016.05.004.
55. Zhang, G.; Schetter, A.; He, P.; Funamizu, N.; Gaedcke, J.; Ghadimi, B.M.; Ried, T.; Hassan, R.; Yfantis, H.G.; Lee, D.H.; et al. DPEP1 inhibits tumor cell invasiveness, enhances chemosensitivity and predicts clinical outcome in pancreatic ductal adenocarcinoma. *PLoS ONE* **2012**, *7*, e31507, doi:10.1371/journal.pone.0031507.
56. Winter, C.; Kristiansen, G.; Kersting, S.; Roy, J.; Aust, D.; Knosel, T.; Rummele, P.; Jahnke, B.; Hentrich, V.; Ruckert, F.; et al. Google goes cancer: Improving outcome prediction for cancer patients by network-based ranking of marker genes. *PLoS Comput. Biol.* **2012**, *8*, e1002511, doi:10.1371/journal.pcbi.1002511.
57. Cancer Genome Atlas Research Network. Electronic address, a.a.d.h.e.; Cancer Genome Atlas Research, N. Integrated Genomic Characterization of Pancreatic Ductal Adenocarcinoma. *Cancer Cell* **2017**, *32*, 185–203 e113, doi:10.1016/j.ccell.2017.07.007.
58. Stratford, J.K.; Bentrem, D.J.; Anderson, J.M.; Fan, C.; Volmar, K.A.; Marron, J.S.; Routh, E.D.; Caskey, L.S.; Samuel, J.C.; Der, C.J.; et al. A six-gene signature predicts survival of patients with localized pancreatic ductal adenocarcinoma. *PLoS Med.* **2010**, *7*, e1000307, doi:10.1371/journal.pmed.1000307.
59. Chen, D.T.; Davis-Yadley, A.H.; Huang, P.Y.; Husain, K.; Centeno, B.A.; Permuth-Wey, J.; Pimiento, J.M.; Malafa, M. Prognostic Fifteen-Gene Signature for Early Stage Pancreatic Ductal Adenocarcinoma. *PLoS ONE* **2015**, *10*, e0133562, doi:10.1371/journal.pone.0133562.

© 2020 by the authors. Submitted for possible open access publication under the terms and conditions of the Creative Commons Attribution (CC BY) license (http://creativecommons.org/licenses/by/4.0/).
